# Quality, physicochemical, and textural properties of dairy products containing fruits and vegetables: A review

**DOI:** 10.1002/fsn3.2430

**Published:** 2021-06-24

**Authors:** Fakhreddin Salehi

**Affiliations:** ^1^ Faculty of Agriculture Bu‐Ali Sina University Hamedan Iran

**Keywords:** cheese, color, fiber, ice cream, yogurt

## Abstract

Due to the high utilization rate of dairy products, enrichment of these products will successfully decrease or prevent diseases related with nutrition deficiencies. Fruits and vegetables in different forms (i.e., fresh, juices, powder, puree, and extract) are excellent sources for the enrichment of dairy products because of their desired taste, color, aroma, fibers, and vitamins content. So, this manuscript reviews the effect of some fruits and vegetables on the rheological behavior, physicochemical attributes, color parameters, sensorial and quality properties of dairy products including cheeses, ice creams, and yogurts. The physicochemical, color, texture, and sensorial properties of dairy products were affected with addition of fruits or vegetables. Also, the addition of these products contributes to the higher content of vitamins, natural colorants, minerals, polyphenols, crude fiber, and carotenoids. In addition, some fruits and vegetables are considered as potential dairy products stabilizing agent due to their desirable functional properties, such as water binding and holding, gelling and thickening ability. In summary, enrichment of cheeses, ice creams, and yogurts with fruits and vegetables increase the market share of these products due to the high demand for goods for an improved diet, rich in compounds with antioxidant activity and biological properties.

## Graphical abstract

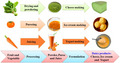

## 1 INTRODUCTION

**FIGURE 1:**
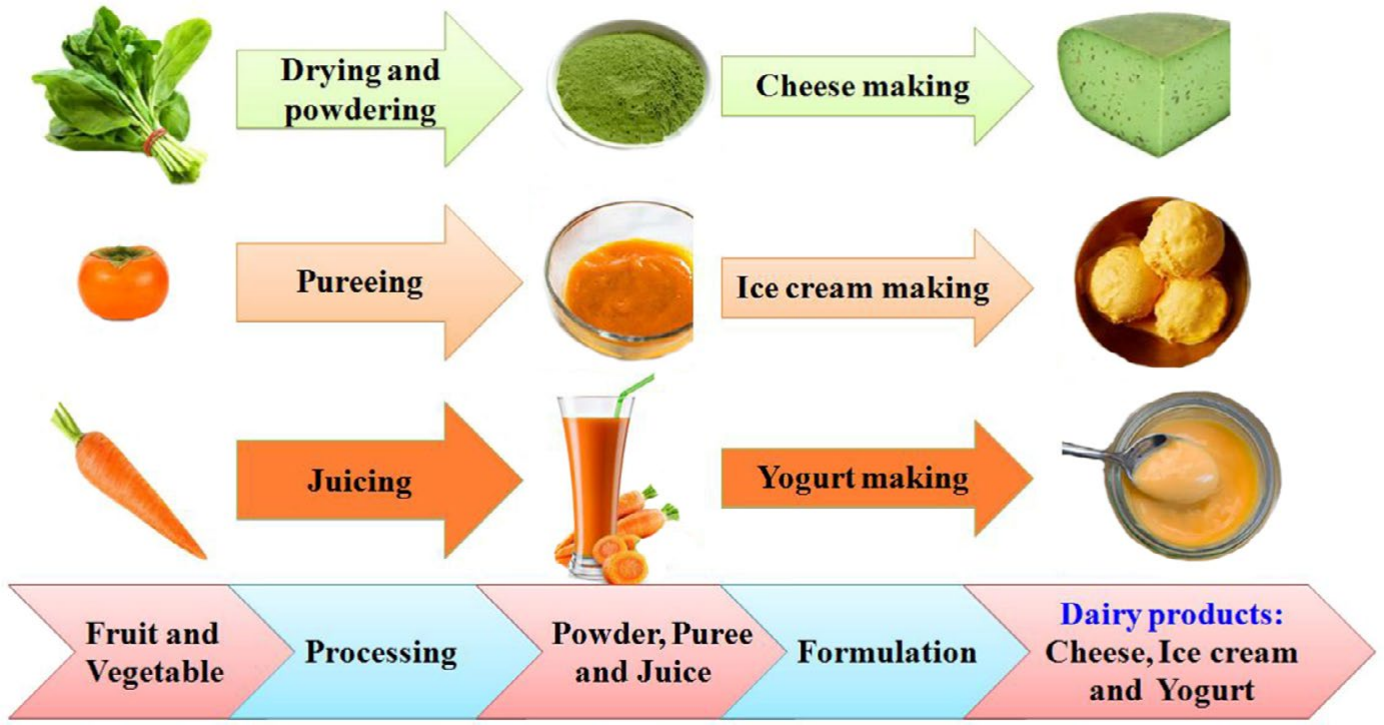
Schematic of fruits and vegetables used for the formulation of dairy products

Researchers and food manufactures are interested in study, practice, and produce that can develop the nutrient profile of food products. Milk is considered as a healthy product with substantial health benefits. Dairy products provide a unique package of high essential nutrients (proteins, fatty acids, vitamins, and minerals) that are important for healthy blood, nervous and immune systems, eyesight, muscle and nerve function, healthy skin, energy levels, and growth and repair in all parts of the body. They can also be considered an excellent matrix for the release of bioactive compounds. In addition, these products can improve health or well‐being and, when consumed at recommended levels, their benefits include improved immune system function, reduced risk of cardiovascular, reduced risk of bone mass loss, and protection against free radical damage (Gebreyowhans et al., 2019; McCain et al., 2018; Verruck et al., 2019). Fruits and vegetables in different forms (i.e., fresh, dried, powder, juices, puree, pulp, fiber, and extract) provide means for producers to improving the health benefits of food products (Salehi, 2020f; Salehi & Aghajanzadeh, 2020; Salehi & Satorabi, 2021; Satorabi et al., 2021). Addition of these products or their by‐products in dairy products including yogurts, ice creams, and cheeses has been studied (Dibazar et al., 2016; Estrada et al., 2011; Granato et al., 2018; Hashemi Gahruie et al., 2015; Jeong et al., 2017; Sah et al., 2016; Wang et al., 2019). Figure 1 shows the schematic of some fruits and vegetables used for the formulation of cheeses, ice creams, and yogurts. For example, spinach powder, persimmon puree, and carrot juice could be used to improving the nutritional value and quality of cheese, ice cream, and yogurt, respectively.

The effect of fruits and vegetables on different characteristics of some dairy products is reported in Table 1. Data listed in this table show the physicochemical attributes of cheeses, ice creams, and yogurts containing fruits and vegetables powder. In summary, enrichment of cheeses, ice creams, and yogurts with fruits and vegetables provides functional dairy products with high nutritional values and acceptability.

**TABLE 1:** Effect of fruits and vegetables on different characteristics of dairy products

Dairy product	Fruit or vegetable	Incorporated concentrations	Suggested percent	Results	References
Cheese	Carrot paste	5%, 10%, and 15%	15%	The use of carrot paste in cheese improves its quality, antioxidant activity, vitamin A content, and Na/K ratio compared with the control sample.	Mohamed et al., (2016)
Cheese	Carrot paste	0%, 10%, 20%, 30%, 40%, and 50%	30%	Carrot addition in chhana (analogous to cottage cheese) reduces the rate of acid formation, decreases the free fatty acid formation and the absorptivity of Rasgulla (sweet syrupy cheese ball) samples, and gives color stability.	Bandyopadhyay et al., (2008)
Cheese	Broccoli	3%, 5%, 10%, 15%, 20%, 30%, and 50%	20%	All broccoli‐cheese powder blended samples contained more total polyphenols as compared to the plain conventional cheese powder.	Sharma et al., (2011)
Cheese	Grape extracts	0%, 0.1%, 0.2%, and 0.3%	‐	Syneresis of milk gels reduced with rising of grape extracts concentrations, that resulted in cheese with high moisture content.	Da Silva et al., (2015)
Cheese (semi‐hard)	Grape pomace	0.8% and 1.6%	1.6%	With addition of grape pomace powders to the cheeses formulation, antioxidant activity and phenolic content in all enriched samples were increased.	Marchiani et al., (2016)
Cheese	Sesame	1%, 3% and 5%	3%	The cheese product was found to have the best sensory characteristics at the 3% sesame seed dose compared with the doses of 1% and 5%.	Slozhenkina et al., (2019)
Cheese (UF‐Soft)	Spinach powder	0.5%, 1%, 1.5%, and 2%	0.5% and 1%	The total solid, protein contents, and acidity of cheese samples were increased considerably with the increasing of spinach nano‐powder concentration.	El‐Sayed, (2020)
Cheese (Queso Blanco)	Tomato extracts	0.5%, 1%, 1.5%, and 2%	2%	Sensorial evaluation scores for the yellowness, tomato taste, and hardness of cheese were considerably higher after fortification with tomato extracts (powdered microcapsules).	Jeong et al., (2017)
Fermented goat milk	Strawberry juice	15%	15%	All samples showed improved fruit aroma and decreased goaty flavor.	Wang et al., (2019)
Ice cream	Cape gooseberry	5%, 10%, and 15%	15%	With increasing cape gooseberry content in ice cream formulation, the values of protein, fat, Ca and P reduced; But, the K, Fe, and Zn contents of samples were increased.	Erkaya et al., (2012)
Ice cream	Carotenoids (extracted from tomato peel)	1%, 2%, 3%, 4%, and 5	2% and 3%	Ice cream containing 2% and 3% of carotenoids (lyco‐red) had the highest scores for flavor, body and textural properties, melting and color, and the best mix.	Rizk et al., (2014)
Ice cream	Citrus fiber	0%.4%, 0.8%, and 1.2%	0.8%	A mixture of citrus fiber and stabilizer/emulsifier created pleasant ice cream characteristics.	Dervisoglu and Yazici, (2006)
Ice cream	Hazelnut flour (HF); hazelnut kernel skin (HKS)	HF: 1.5%, 3%, and 4.5% HKS: 1%, 2%, and 3%	3% HF and 1% HKS	The ice cream with HF showed high pH, nitrogen, ash, apparent viscosity, and L*, flavor, body and texture, and appearance values than the samples with HKS.	Dervisoglu, (2006)
Ice cream	Kiwifruit	49%	49%	The ice creams kept the polyphenols and vitamin C contents in addition the natural color flavor of the kiwifruit added.	Sun‐Waterhouse et al., (2013)
Ice cream	Orange by‐products fiber	0.74%	0.74%	Orange by‐products fiber proved to be a promising alternative as a fat replacer in ice cream production.	De Moraes Crizel et al., (2013)
Ice cream	Orange fiber	1.0% and 1.5%	1.0%	The orange fiber can be utilized to decrease the fat content (50%) and increase bioactive compounds content, such as dietary fibers and carotenoids.	Crizel et al., (2014)
Ice cream	Persimmon	8%, 16%, 24%, 32%, and 40%	24%	Better melting characteristics and texture parameters were reported for the ice creams containing different concentrations of persimmon puree.	Karaman et al., (2014)
Ice cream	Quince seed powder	0%, 0.25%, 0.5%, and 0.75%	0.75%	The adding of quince seed powder improved the protein content compared with the control samples due to the high protein content of quince seed powder (35%). The fortification of ice cream with quince seed powder can make an effective way to improve the nutritional and structural properties of ice creams.	Kurt and Atalar, (2018)
Ice cream	Strawberry	15%, 20%, or 25%	25%	In strawberry ice cream‐type frozen yogurt, the structure hardened in parallel with the enhancement in strawberry percent.	Guven and Karaca, (2002)
Ice cream	Tea or herbal teas	2.5% and 5.0%	‐	Total phenolic content of ice cream samples increased with the addition of tea or herbal teas (sage, chamomile, and linden) and it was in the range of 123.4–415.2 mg/kg.	Karaman and Kayacier, (2012)
Yogurt	Apple, banana, or passion fruit processing by‐products	‐	‐	These fruit dietary fibers can improve the fatty acid profile of probiotic yogurts and point out the suitability of using dietary fibers from fruit processing the by‐products to develop high value‐added fermented dairy products.	Do Espírito Santo et al., (2012)
Yogurt	Apple, wheat, bamboo and inulin fibers	1.3%	1.3%	Syneresis and pH did not show any difference, while only apple dietary fibers yogurt showed color differences compared to the control sample. The adding of 1.3% dietary fibers to the formulation of yogurts appears to be a promising avenue for increased dietary fibers intake, with high user satisfactoriness.	Staffolo et al., (2004)
Yogurt	Apple pomace	1%, 2%, and 3%	3%	Apple pomace (3%) decreased the syneresis of stirred yogurt by half that of the control. Also, it contributed to total phenolics and dietary fibers to stirred yogurt.	Wang et al., (2020)
Yogurt	Apple pomace	0.1%, 0.5%, and 1%	1%	Apple pomace induced earlier milk gelation and shorter fermentation time, especially at the 1% level.	Wang, Kristo, et al., (2019)
Yogurt	Apple pomace fiber	0%, 2.5%, 5%, 7.5%, and 10%	5%	The addition of dietary fibers in yogurt resulted in a reduction of acidity (0.15%–0.09%) and fat contents (1.65%–1.59%) with the increase in fiber concentration.	Issar et al., (2017)
Yogurt	Carrot juice	0%, 10%, 15%, and 20%	10%–15%	Total yogurt carotenoid content was considerably improved with carrot juice enrichment.	Kiros et al., (2016)
Yogurt	Carrot cell wall particles	0%, 0.5%, 1%, 1.5% and 2%	‐	The improved gel strength and decreased whey loss achieved by the addition of cell wall particles were maintained throughout the 28‐day storage period.	McCann et al., (2011)
Yogurt	Date fiber	1.5%, 3%, and 4.5%	3%	Fortifying yogurt with 3% date fiber produced acceptable yogurt with beneficial health property. Yogurts fortified with date fiber had firmer texture and darker color (lower L* and higher a*) compared with control yogurt.	Hashim et al., (2009)
Yogurt	*Euterpe oleracea* juice	10%	10%	The authors suggest that *E. oleracea* juice can be used as a natural functional pigment for flavoring and coloring yogurt.	Coïsson et al., (2005)
Yogurt	Grape fiber	0%–1.2%	0.9%	The moisture and syneresis of fruit yogurts notably decreased by increasing grape fibers amount.	Dibazar et al., (2016)
Yogurt	Wine grape pomace	2%	2%	The enriched products had increased dietary fibers and polyphenols contents and had delayed lipid oxidation during refrigeration storage. Also, it was acceptable by consumers based on the sensorial analysis.	Tseng and Zhao, (2013)
Yogurt	Orange fiber	0%, 0.6%, 0.8%, and 1%	1%	Yogurts with 1% fiber were considerably various from the others along with cold storage, presenting lower L*, higher a* and b* values, and lower syneresis.	García‐Pérez et al., (2005)
Yogurt	Orange fiber	0%, 0.2%, 0.4%, 0.6%, 0.8% and 1%	‐	Elastic moduli (G′), viscous moduli (G″), and apparent viscosity were higher in yogurts with large particles than in yogurts with the dietary fibers of smaller size.	Sendra et al., (2010)
Yogurt	Passion fruit fiber	1%	1%	Thixotropy of supplemented yogurts was higher than that of their respective controls in the two cycles of shear rate.	Espírito‐Santo et al., (2013)
Yogurt	Pineapple peel powder	1.16%	1.16%	Pineapple peel powder addition to yogurt lowered firmness and storage modulus. Syneresis level in probiotic yogurt with pineapple peel powder (1.2% at day 1) was comparable with the prebiotic‐inulin and increased during storage.	Sah et al., (2016)
Yogurt	Pumpkin fiber	0.5%, 1.0%, and 1.5%	1%	Pumpkin fiber could improve the physical quality and contributed to textural characteristics of half‐fat yogurt.	Bakirci et al., (2017)
Yogurt	Strawberry pulp	15% and 30%	30%	The addition 30% of strawberry cryoconcentrates resulted in a product with higher anthocyanins content and antioxidant activity.	Gasparrini et al., (2017)
Yogurt	Strawberry	10%–20%	‐	The addition of strawberry pieces to yogurt can decrease free polyphenols and whey protein contents, possibly constraining its bioaccessibility in yogurt.	Oliveira et al., (2015)
Yogurt	Strawberry	13%	‐	Apparent viscosity of the flavored yogurts considerably increased during the storage periods (7, 14, and 28 days, at 10°C).	Lubbers et al., (2004)
Yogurt	Yam soluble fiber	1%	1%	Incorporation of the yam soluble fiber significantly decreased the syneresis. It produced a more acceptable mouthfeel in the fortified yogurt in comparison to the control samples, indicating the viability of the process to obtain a marketable product.	Ramirez‐Santiago et al., (2010)

Due to the high utilization rate of dairy products, enrichment of these products will successfully decrease or prevent diseases related with nutrition deficiencies (Gebreyowhans et al., 2019). Dairy products are rich in protein, fatty acids, calcium, potassium, B vitamins but are deficient in iron, vitamin C, carotenes, and dietary fibers (Caleja et al., 2016; Carocho et al., 2015; Hashemi Gahruie et al., 2015). Thus, the combination of fruits and vegetables products and by‐products and cheeses, ice creams, and yogurts will improve the nutritional and functional food characteristics of these products. Vitamins, polyphenols, and carotenoids are considered the most natural antioxidant molecules in fruits and vegetables. These natural compounds can provide better sensory, nutrition, and antioxidant quality compared to the artificial additives in dairy products (Caleja et al., 2016).

Fruits and vegetables products could be used to enhance the fiber content of dairy products. The diet with a high amount of fibers is reported to have an encouraging influence on health (Salehi & Aghajanzadeh, 2020). The significance of food fibers has led to the improvement of a high and potential market for fiber‐rich products and components. Newly, there are trends to find a new source of dietary fibers that could be used in the dairy products. Fruits and vegetables fibers have better quality due to higher total and soluble fiber and water content and oil holding capacity and colonic ferment ability, in addition to lower phytic acid content and caloric value (Issar et al., 2017; Salehi, 2017). Also, the primary dietary source of phenolic compounds is present in fruits and vegetables products. It has been suggested that these products extracts, juices, and powders have the potential to be applied as functional ingredients in dairy products (Coïsson et al., 2005; Karaaslan et al., 2011; Salehi, 2020d; Wallace & Giusti, 2008).

Food enrichment is one of the most important processes for the enhancement of the nutritional quality and quantity in food products. Currently, the development of new dairy products formulations that are highly enjoyed by consumers is one of the driving forces of dairy industries. Fruits and vegetables are rich sources of total phenol, antioxidant, carotenes, minerals, and dietary fiber, and could enrich dairy products with these compounds. Fortified cheeses, ice creams, and yogurts appeal to a wide range of consumers and have the potential to enhance sales in the dairy industry. So, the present study summarized the effect of some fruits and vegetables includes apple, broccoli, carrot, grape, grapefruit, kiwifruit, orange, persimmon, pineapple, pumpkin, spinach, strawberry, and tomato on the physicochemical, color, textural, and sensorial properties and quality of dairy products, including cheeses, ice creams, and yogurts.

## 2 CHEESE

Cheese is one of the mainly popular dairy products, that is a rich source of necessary nutrients such as vitamins, amino acids, and minerals (López‐Expósito et al., 2012). Also, consumers worldwide demand the development of cheese with reduced levels of synthetic additives, such as flavoring and coloring agents. In this section, I listed a plethora of applications of fruits and vegetables products in cheeses. Some researchers have studied the enrichment of cheeses by different nutrients and various sources of dietary fibers (El‐Sayed, 2020; Mohamed et al., 2016). The physicochemical properties of the cheeses samples that were enriched with fruits and vegetables at the various concentrations are shown in Table 2.

**TABLE 2:** Physicochemical attributes of dairy products containing fruits or vegetables

Dairy product	Fruit or vegetable	Moisture (%)	Fat (%)	Protein (%)	Total solid (%)	Fiber (%)	pH	Acidity	Syneresis (%)	Ash (%)	β‐Carotene (mg/kg)	Total phenolic (µg gallic acid equivalent/g)	References
Cheese	Broccoli: 3%	‐^a^	‐	‐	‐	‐	‐	‐	‐	‐	15.9	3,389.9	Sharma et al., (2011)
	Broccoli: 5%	‐	‐	‐	‐	‐	‐	‐	‐	‐	21.2	3,866.9	
	Broccoli: 10%	‐	‐	‐	‐	‐	‐	‐	‐	‐	35.6	3,950.6	
	Broccoli: 15%	‐	‐	‐	‐	‐	‐	‐	‐	‐	44.3	4,589.5	
	Broccoli: 20%	‐	‐	‐	‐	‐	‐	‐	‐	‐	59.8	4,504.2	
	Broccoli: 30%	‐	‐	‐	‐	‐	‐	‐	‐	‐	104.3	4,146.8	
	Broccoli: 50%	‐	‐	‐	‐	‐	‐	‐	‐	‐	140.4	4,239.1	
Cheese	Carrot paste: 0%	‐	‐	14.66	39.75	‐	5.70	‐	‐	4.02	‐	150	Mohamed et al., (2016)
	Carrot paste: 5%	‐	‐	12.18	40.78	0.45	5.78	‐	‐	3.25	15.6	1,491	
	Carrot paste: 10%	‐	‐	12.01	40.82	0.63	5.85	‐	‐	3.82	22.16	1,512	
	Carrot paste: 15%	‐	‐	11.55	40.90	0.69	5.88	‐	‐	3.34	29.23	1,539	
Cheese (UF‐Soft)	Spinach powder: 0%	68.84	13.0	9.29	31.16	‐	6.36	0.14	‐	2.39	‐	‐	El‐Sayed, (2020)
	Spinach powder: 0.5%	68.65	13.0	9.50	31.35	0.15	6.30	0.14	‐	2.50	‐	‐	
	Spinach powder: 1%	68.32	12.5	9.83	31.68	0.27	6.28	0.16	‐	2.61	‐	‐	
	Spinach powder: 1.5%	68.02	12.5	9.91	31.98	0.38	6.25	0.18	‐	2.75	‐	‐	
	Spinach powder: 2%	67.75	11.5	10.03	32.25	0.51	6.20	0.20	‐	2.93	‐	‐	
Cheese (Queso Blanco)	Tomato extracts: 0%	47.45	20.58	26.39	‐	‐	5.64	‐	‐	2.56	‐	‐	Jeong et al., (2017)
	Tomato extracts: 0.5%	47.30	20.73	26.38	‐	‐	5.64	‐	‐	2.56	‐	‐	
	Tomato extracts: 1%	46.87	20.60	26.73	‐	‐	5.62	‐	‐	2.59	‐	‐	
	Tomato extracts: 1.5%	46.03	20.68	26.61	‐	‐	5.42	‐	‐	2.60	‐	‐	
	Tomato extracts: 2%	46.23	20.54	26.44	‐	‐	5.24	‐	‐	2.57	‐	‐	
Ice cream	Cape gooseberry: 0%	70.69	4.60	5.80	29.31	‐	6.30	9.72°SH^b^	‐	0.82	‐	‐	Erkaya et al., (2012)
	Cape gooseberry: 5%	68.66	4.13	5.37	31.43	‐	6.13	12.98°SH	‐	0.89	‐	‐	
	Cape gooseberry: 10%	66.47	3.88	4.78	33.53	‐	5.98	14.24°SH	‐	0.91	‐	‐	
	Cape gooseberry: 15%	64.8	3.70	3.85	35.20	‐	5.83	14.66°SH	‐	0.95	‐	‐	
Ice cream	Carotenoids: 0%	‐	8.00	4.60	32.93	‐	6.22	‐	‐	1.10	‐	‐	Rizk et al., (2014)
	Carotenoids: 1%	‐	8.13	4.61	32.92	‐	6.20	‐	‐	1.10	‐	‐	
	Carotenoids: 2%	‐	8.25	4.60	32.91	‐	6.16	‐	‐	1.11	‐	‐	
	Carotenoids: 3%	‐	8.33	4.61	32.91	‐	6.14	‐	‐	1.12	‐	‐	
	Carotenoids: 4%	‐	8.45	4.62	32.91	‐	6.10	‐	‐	1.12	‐	‐	
	Carotenoids: 5%	‐	8.58	4.62	32.90	‐	6.07	‐	‐	1.13	‐	‐	
Ice cream	Citrus fiber: 0%	65.12	7.43	‐	34.88	‐	6.16	0.24	‐	0.87	‐	‐	Dervisoglu and Yazici, (2006)
	Citrus fiber: 0.4%	64.69	7.03	‐	35.31	‐	6.30	0.23	‐	0.78	‐	‐	
	Citrus fiber: 0.8%	64.64	7.03	‐	35.36	‐	6.26	0.24	‐	0.87	‐	‐	
	Citrus fiber: 1.2%	64.61	6.87	‐	35.39	‐	6.25	0.24	‐	0.96	‐	‐	
Ice cream	Orange fiber: 0%	63.03	18.52	12.87	‐	‐	‐	‐	‐	3.41	‐	‐	De Moraes Crizel et al., (2013)
	Orange fiber: 0.74%	69.26	5.32	12.48	‐	‐	‐	‐	‐	3.69	‐	‐	
Ice cream	Orange fiber: 0%	‐	8.24	‐	‐	0	‐	‐	‐	‐	‐	‐	Crizel et al., (2014)
	Orange fiber: 1%	‐	4.10	‐	‐	0.64	‐	‐	‐	‐	‐	‐	
	Orange fiber: 1.5%	‐	3.84	‐	‐	0.88	‐	‐	‐	‐	‐	‐	
Ice cream	Control	65.36	6.5	3.62	34.64	‐	‐	‐	‐	‐	‐	‐	Akalın et al., (2018)
	2% apple fiber	63.27	6.0	4.05	36.73	‐	‐	‐	‐	‐	‐	‐	
	2% orange fiber	62.69	6.0	4.02	37.31	‐	‐	‐	‐	‐	‐	‐	
	2% oat fiber	62.48	6.0	3.76	37.52	‐	‐	‐	‐	‐	‐	‐	
	2% bamboo fiber	63.2	6.0	3.76	36.8	‐	‐	‐	‐	‐	‐	‐	
	2% wheat fiber	63.23	6.0	3.76	36.77	‐	‐	‐	‐	‐	‐	‐	
Ice cream	Persimmon puree: 0%	‐	9.03	4.66	32.51	‐	6.66	‐	‐	1.08	‐	‐	Karaman et al., (2014)
	Persimmon puree: 8%	‐	8.31	4.25	31.62	‐	6.76	‐	‐	1.03	‐	‐	
	Persimmon puree: 16%	‐	7.58	3.77	30.62	‐	6.91	‐	‐	0.97	‐	‐	
	Persimmon puree: 24%	‐	6.86	3.45	29.84	‐	7.00	‐	‐	0.91	‐	‐	
	Persimmon puree: 32%	‐	6.14	3.31	28.84	‐	6.93	‐	‐	0.84	‐	‐	
	Persimmon puree: 40%	‐	5.41	2.63	28.02	‐	6.76	‐	‐	0.78	‐	‐	
Ice cream	Quince seed powder: 0%	‐	3.05	3.04	28.38	‐	6.65	‐	‐	1.98	‐	‐	Kurt and Atalar, (2018)
	Quince seed powder: 0.25%	‐	3.10	3.24	28.40	‐	6.65	‐	‐	1.94	‐	‐	
	Quince seed powder: 0.05%	‐	3.05	3.29	30.03	‐	6.63	‐	‐	2.06	‐	‐	
	Quince seed powder: 0.75%	‐	3.05	3.48	30.17	‐	6.62	‐	‐	2.08	‐	‐	
Yogurt	Apple pomace: 0%	‐	‐	‐	‐	‐	‐	‐	30.0	‐	‐	52.0	Wang et al., (2020)
	Apple pomace: 1%	‐	‐	‐	‐	‐	‐	‐	23.7	‐	‐	68.3	
	Apple pomace: 2%	‐	‐	‐	‐	‐	‐	‐	19.5	‐	‐	84.6	
	Apple pomace: 3%	‐	‐	‐	‐	‐	‐	‐	16.3	‐	‐	91.3	
Yogurt	Carrot juice: 0%	82.58	3.30	3.14	17.42	‐	‐	0.71%	36.39	0.77	3.05	37.00	Kiros et al., (2016)
	Carrot juice: 10%	83.29	3.00	2.86	16.71	‐	‐	0.68%	42.66	0.76	6.73	36.49	
	Carrot juice: 15%	83.56	2.89	2.79	14.44	‐	‐	0.66%	44.84	0.75	8.71	36.58	
	Carrot juice: 20%	83.85	2.76	2.77	16.15	‐	‐	0.62%	45.33	0.74	10.26	35.75	
Yogurt	Yam soluble fiber: 0%	83.5	2.2	3.1	‐	‐	‐	90.8°D^c^	23.3	‐	‐	‐	Ramirez‐Santiago et al., (2010)
	Yam soluble fiber: 1%	83.1	1.9	2.6	‐	‐	‐	93.0°D	15.4	‐	‐	‐	
Yogurt	Apple pomace fiber: 0.0%	85.01	1.65	‐	7.00	‐	‐	0.15%	‐	1.08	‐	‐	Issar et al., (2017)
	Apple pomace fiber: 2.5%	82.52	1.60	‐	6.90	2.42	‐	0.13%	‐	1.09	‐	‐	
	Apple pomace fiber: 5.0%	81.74	1.60	‐	9.90	4.34	‐	0.12%	‐	1.09	‐	‐	
	Apple pomace fiber: 7.5%	81.68	1.59	‐	6.70	6.97	‐	0.10%	‐	1.10	‐	‐	
	Apple pomace fiber: 10.0%	81.64	1.59	‐	6.50	9.94	‐	0.09%	‐	1.10	‐	‐	
Yogurt	Date fiber: 0%	‐	‐	‐	‐	‐	‐	1.04%	‐	‐	‐	‐	Hashim et al., (2009)
	Date fiber: 1.5%	‐	‐	‐	‐	‐	‐	1.08%	‐	‐	‐	‐	
	Date fiber: 3.0%	‐	‐	‐	‐	‐	‐	1.08%	‐	‐	‐	‐	
	Date fiber: 4.5%	‐	‐	‐	‐	‐	‐	1.07%	‐	‐	‐	‐	
Yogurt	Orange fiber: 0%	79.47	3.03	3.97	‐	‐	‐	‐	‐	1.28	‐	‐	García‐Pérez et al., (2005)
	Orange fiber: 0.6%	80.49	3.33	3.91	‐	‐	‐	‐	‐	0.98	‐	‐	
	Orange fiber: 0.8%	80.66	3.43	3.90	‐	‐	‐	‐	‐	0.98	‐	‐	
	Orange fiber: 1%	79.17	2.95	3.97	‐	‐	‐	‐	‐	1.16	‐	‐	
Yogurt	Pumpkin fiber: 0%	‐	1.73	4.43	13.05	‐	‐	1.23%	‐	‐	‐	‐	Bakirci et al., (2017)
	Pumpkin fiber: 0.5%	‐	1.77	4.70	14.01	‐	‐	1.24%	‐	‐	‐	‐	
	Pumpkin fiber: 1%	‐	1.75	4.73	14.87	‐	‐	1.29%	‐	‐	‐	‐	
	Pumpkin fiber: 1.5%	‐	1.77	4.87	15.16	‐	‐	1.28%	‐	‐	‐	‐	
Yogurt	Strawberry pulp:0%	‐	‐	‐	11.23	‐	‐	0.44%	‐	‐	‐	‐	Jaster et al., (2018)
	Strawberry pulp:15%	‐	‐	‐	12.02	‐	‐	0.69%	‐	‐	‐	‐	
	Strawberry pulp:30%	‐	‐	‐	12.22	‐	‐	1.00%	‐	‐	‐	‐	

^a^ No data available.

^b^ Soxhlet‐Henkel degree (°SH).

^c^ Dornic degree (°D).

It is important to note that nutritional value, safety (microbiological and toxicological), and sensory properties should be taken into consideration when cheese added with fruits and vegetables products is manufactured. As shown in Table 1, the effect of carrot paste (Bandyopadhyay et al., 2008; Mohamed et al., 2016), broccoli (Sharma et al., 2011), grape extracts (Da Silva et al., 2015), grape pomace (Marchiani et al., 2016), sesame (Slozhenkina et al., 2019), spinach powder (El‐Sayed, 2020), and tomato extracts (Jeong et al., 2017) was studied on different properties of the cheese samples. Therefore, in this section, the effects of using different fruits and vegetables on the nutritional values, physicochemical properties, organoleptic aspects, and microbial attributes of the cheeses were reviewed.

Spinach is a good source of minerals (Fe, Mn, Zn, and Mg), vitamins (E, A, C, K, B_1_, B_6_, and B_2_), protein, fibers, and antioxidants, making it a suitable ingredient to be used in the formulation of dairy products with high nutritional and biological values (Slavin & Lloyd, 2012). Sharma et al., (2011) studied the physicochemical and sensorial properties, antioxidant capacity, polyphenols, and pigments of cheese enriched with broccoli powder (freeze dried) and observed that the combined cheese powder containing up to 20% broccoli powder was a suitable product and total antioxidant capacity, polyphenols, and carotenoids of broccoli‐cheese were increased by increasing the broccoli powder concentration. In another study, El‐Sayed (2020) studied the effect of spinach nano‐powder (0.5%–2%) addition on the quality of ultra‐filtered soft cheeses. The author demonstrated that by increasing of spinach powder concentration with retentate, the content of fibers, minerals, total phenolic content, and antioxidant activity of samples was enhanced.

Carrot is a rich source of vitamins (thiamine, riboflavin, and vitamin B‐complex), minerals, and β‐carotene (Salehi, 2018; Salehi et al., 2016). Since carrot is a valuable micronutrient source, so, it could be used in dairy products to enhance vitamin A and mineral contents. Bandyopadhyay et al., (2008) evaluated the effect of carrot paste (10%–50%) on the quality of the Rasgulla (sweet syrupy cheese ball). The authors reported that the use of carrot paste up to a concentration of 30% improves the quality of Rasgulla and increases the oxidative and color stabilities of cheese samples. In another study, Mohamed et al., (2016) investigated the quality properties of an analog processed spreadable cheese made with different concentrations (5%–15%) of carrot paste. The additions of carrot pastes improved the antioxidant activity and increased the nutritional components, vitamin A, carotenoids, and phenolic compounds.

Lycopene is a naturally occurring carotenoid in the tomato products and it is an alternative bioactive compound that can be used to improve dairy ingredients. Jeong et al., (2017) studied the physicochemical characteristics, microbial and sensorial parameters of Queso Blanco cheese enriched with powdered microcapsules containing tomato extracts (0.5%–2%) during storage at 7°C for 60 days. The results of this study demonstrate that powdered microcapsules containing tomato extracts can be successfully used in the production of Queso Blanco cheese, and these supplements will benefit the dairy industry and customer's health. Also, the textural properties of Queso Blanco cheese improved after fortification with the powdered microcapsules.

Da Silva Dannenberg et al., (2016) examined the antimicrobial activity of essential oils extracted from pink pepper tree fruit against *Listeria monocytogenes* in Minas‐type fresh cheese during 30 days of storage at 4°C. The authors reported that essential oils from ripe and mature fruits were more efficient toward *L. monocytogenes* compared to green fruit, and the bacterial growth was decreased by 1.3 logs CFU/g in 30 days. Also, their results show that these essential oils have the potential to use as a preservative in food. Essential oils are natural aromatic compounds and volatile liquids that might be derived from flowers, leaves, seeds, peel, fruits, and wood of the plants. Some plants extract and essential oils have demonstrated inhibitory activity against pathogens and spoilage bacteria, even in complex food matrixes such as cheeses (Khorshidian et al., 2018). Potential utilize of passion fruit (PF) as a biopreservative in the making of coalho cheese was studied (Costa et al., 2020). The results indicated the inhibitory of PF and its potential use for controlling microbial populations in cheeses.

Different studies emphasize that fruits and vegetables by‐products can be a rich source of carotenoids, fibers, polyphenols, tocopherols, vitamins, and bioactive compounds (Salehi, 2020b, 2020c). Generally, the use of these by‐products in dairy can improve the quality and nutritional properties, decreases the absorption of cholesterol, reduces hypertension, gastrointestinal disorders, coronary heart disease, and diabetes (Abou‐Zeid, 2016; Hashemi Gahruie et al., 2015), and can also enhance some industrial aspects such as yield, by weight and hardness (Karaman et al., 2014; Kurt & Atalar, 2018). Furthermore, the addition of plant‐based antioxidants in dairy products has met acceptance for the retardation of oxidation in these products (Abou‐Zeid, 2016; Alenisan et al., 2017). Da Silva et al., (2015) examined the effect of commercial grape extracts (from whole grape, grape seed, and grape skin) on the cheese‐making characteristics of milk. With whole grape or grape seed extracts were added to milk at 0.1%, the recovery coefficient for polyphenols was about 0.63 and reduced with increasing extract levels in milk. Also, the addition of grape extracts in milk slightly improved protein recovery in cheeses. In another study, Costa et al., (2018) examined the effect of powders from different by‐products addition (red and white wine grape pomace, tomato peel, broccoli, and artichokes by‐products), at two concentrations of 5% and 10%, on the sensorial and physicochemical properties of Primosale cheese. The authors reported that the addition of these by‐products to Primosale cheese enhanced the nutritional attributes and some sensorial properties (friability and adhesiveness). Also, the addition of these powders at both concentrations (5% and 10%) significantly increased the antioxidant activity and total phenolic content. The aim of Paximada et al., (2021) study was to investigate the possibility of producing low‐fat cheddar cheeses using double emulsions, enriched with milk or vegetable proteins (pumpkin seed protein and rice protein). The cheese with the double emulsions showed lower hardness, and oil loss compared to the low‐fat cheddar cheeses.

## 3 ICE CREAM

Ice cream, a sweet dairy product including milk, sweetener, stabilizer, emulsifier, and flavoring, is produced by mixing its ingredients, followed by pasteurization and homogenization. Afterward, it is aged at low temperature and finally frozen. Some parameters including rich sweet flavor, smooth and good texture, and cold sensation make ice cream a favored product of most people (Bahramparvar et al., 2014; Bahram‐Parvar et al., 2017; Karaman et al., 2014).

Fruits and vegetables are excellent sources for the enrichment of food products because of their sweet and desired taste, color, and aroma (Salehi, 2020a, 2020e). Many studies are reported in the literature about the development of new formulations of ice cream containing fruits and vegetables products. As shown in Table 1, the effect of cape gooseberry (Erkaya et al., 2012), carotenoids (extracted from tomato peel) (Rizk et al., 2014), citrus fiber (Dervisoglu & Yazici, 2006), hazelnut flour ( and hazelnut kernel skin (Dervisoglu, 2006), kiwifruit (Sun‐Waterhouse et al., 2013), orange by‐products fiber (De Moraes Crizel et al., 2013), orange fiber (Crizel et al., 2014), persimmon (Karaman et al., 2014), quince seed powder (Kurt & Atalar, 2018), and strawberry (Guven & Karaca, 2002) were studied on different properties of the ice creams. Therefore, in this section, the effects of using different fruits and vegetables on the nutritional values, physicochemical properties, organoleptic aspects, and microbial attributes of the ice creams were reviewed.

The ice creams available in markets are generally deficient in vitamins, natural antioxidants, colors, and polyphenols. Therefore, enrichment of these products using nutritional ingredients with healthy benefits (focus on natural colorants and antioxidants, vitamins (A, E, B_1_, B_2_, B_6_, and C), minerals, low fat, and freedom from synthetic additives) was studied by some researchers (De Moraes Crizel et al., 2013; Dervisoglu, 2006; Dervisoglu & Yazici, 2006; Erkaya et al., 2012; Favaro‐Trindade et al., 2007; Guven & Karaca, 2002; Karaman & Kayacier, 2012; Karaman et al., 2014). For example, Sun‐Waterhouse et al., (2013) examined the manufacturing ice cream using a considerable amount of juices (49% v/v) from different kiwifruits flesh (green, gold, or red). The authors reported that the ice creams containing different kiwifruits juices retained the carotenoids, polyphenols and vitamin C contents as well as the natural color flavor of the kiwifruit used. In another study, characterization of the main component of carotenoid pigments (β‐carotene, lycopene, *cis*‐lycopene, phytoene, phytofluene, and lutein) extracted from tomato peels and its uses as natural antioxidants and colorants of ice cream were studied (Rizk et al., 2014). The results were indicating that with increases in the carotenoids (lyco‐red) concentration, the radical scavenging activity and ferric reducing antioxidant power of ice creams increased.

Persimmon is a climacteric fruit with unique sensorial properties and nutritional quality. It is a commonly cultivated and consumed fruit in Asian countries, and China has the most substantial persimmon annual yield in the world. It is a good source of bioactive compounds such as ascorbic acid, antioxidants, tannins, polyphenols, and carotenoids (Denoya et al., 2020; Jiang et al., 2020; Salehi et al., 2017). Fortification of ice cream with persimmon (puree or juice) can improve the quality of the ice creams in terms of nutritional, textural, physicochemical, sensorial, and bioactive (antiradical activity and total phenol) properties. In the Karaman et al., (2014) study, persimmon puree was added into the ice cream mix at various levels (8%–40%) and some physicochemical, textural, bioactive, and sensory characteristics of samples were measured. The authors reported that the dry matter, total ash, fat content, protein content, and apparent viscosity of ice creams were decreased with increasing persimmon puree concentration. Also, the glucose, fructose, sucrose, and lactose were characterized to be significant sugars in the ice cream samples, including persimmon and an increase in persimmon puree percent improved the fructose and glucose content.

Enrichment of ice cream with different sources of dietary fibers and phenolic compounds has been the topic of some researches (Saremnezhad et al., 2020; Soukoulis et al., 2009). By‐products of orange juice extraction, as a source of dietary fiber and fat replacer, were added in the formulation of ice cream by De Moraes Crizel et al., (2013). The results indicated the lowest caloric value and improvement of textural properties in those produced ice creams. Also, the ice cream made with the Orange by‐products fiber had a 70% reduction in fat content without causing significant changes in product characteristics such as color, odor, and texture. Soukoulis et al., (2009) investigated the effects of oat, wheat, apple, and inulin fibers on the rheological behavior and thermal characteristics of ice creams. High content in insoluble fibers led to the reinforcement of the viscosity and thixotropy of ice cream mixes. Their results suggest the potential utility of dietary fiber as crystallization and recrystallization phenomena controller in ice creams. In another study, Akalın et al., (2018) evaluated the effect of five dietary fibers including orange, apple, oat, bamboo, and wheat on the quality characteristics, physicochemical, rheological behavior, textural and sensorial properties of probiotic ice creams. The authors reported that the addition of wheat fiber in ice cream improved rheological behavior, maintained sensorial properties, and kept probiotics viable during storage. In contrast, ice creams samples with orange and apple fibers had better melting resistance. The composition and the soluble to insoluble ratio are significant parameters for the demonstrated functionality of the additional dietary fibers.

Understanding fat, proteins, and saliva impact on aroma release from flavored ice creams was studied by Ayed et al., (2018). At low fat level more aroma compounds were released from ice creams with lower protein content. The color parameters, textural properties, and apparent viscosity of some dairy products containing fruits and vegetables are summarized in Table 3. The addition of orange and apple dietary fibers decreased the lightness (L*) value, whereas the ice cream samples with wheat, oat, and bamboo did not considerably vary from the control sample in terms of that L*. Also, the presence of apple and orange fibers improved the red and yellow colors of the ice cream, resulting in increasing in the values of a* (redness) and b* (yellowness), respectively (Akalın et al., 2018). Orange fibers were used as a new fat replacer in light lemon ice cream (Crizel et al., 2014). The ice cream samples supplemented with orange fiber had considerably lower L* values and higher a* and b* coordinate values, indicating that the use of these fibers resulted in a product that was less bright and more red and yellow than the others. In addition, the orange fiber decreased about 50% in ice cream fat content and it increased fiber content and some textural characteristics values (firmness, gumminess and springiness), but it did not influence the adhesiveness and odor of the ice cream samples. The total acceptance of the ice cream with 1% of pretreated orange peel fiber did not vary from that of the control sample (80%).

**TABLE 3:** Textural and rheological properties, and color parameters of dairy products containing fruits or vegetables

Dairy product	Fruit or vegetable	Firmness	Consistency (N.s)	Cohesiveness	Chewiness (J)	Adhesiveness (g.s)	Springiness	Viscosity (mPa·s)	L^*a^	a^*^	b^*^	References
Cheese (UF‐Soft)	Spinach powder: 0%	‐^b^	‐	‐	‐	‐	‐	‐	70.05	−1.17	21.49	El‐Sayed, (2020)
	Spinach powder: 0.5%	‐	‐	‐	‐	‐	‐	‐	68.33	−1.89	19.45	
	Spinach powder: 1%	‐	‐	‐	‐	‐	‐	‐	65.16	−2.11	17.58	
	Spinach powder: 1.5%	‐	‐	‐	‐	‐	‐	‐	57.67	−2.56	16.94	
	Spinach powder: 2%	‐	‐	‐	‐	‐	‐	‐	51.85	−2.68	14.09	
Cheese (Queso Blanco)	Tomato extracts: 0%	14.36 N^c^	‐	0.71	8.52	‐	0.71 mm	‐	81.10	−4.21	16.70	Jeong et al., (2017)
	Tomato extracts: 0.5%	15.83 N	‐	0.71	10.11	‐	0.72 mm	‐	77.70	1.15	23.75	
	Tomato extracts: 1%	16.18 N	‐	0.72	10.20	‐	0.73 mm	‐	76.93	1.47	25.74	
	Tomato extracts: 1.5%	19.14 N	‐	0.73	11.78	‐	0.83 mm	‐	75.49	3.03	28.50	
	Tomato extracts: 2%	19.18 N	‐	0.73	13.36	‐	1.00 mm	‐	74.51	3.50	29.84	
Ice cream	Cape gooseberry: 0%	‐	‐	‐	‐	‐	‐	1,714	‐	‐	‐	Erkaya et al., (2012)
	Cape gooseberry: 5%	‐	‐	‐	‐	‐	‐	2,161	‐	‐	‐	
	Cape gooseberry: 10%	‐	‐	‐	‐	‐	‐	3,430	‐	‐	‐	
	Cape gooseberry: 15%	‐	‐	‐	‐	‐	‐	4,654	‐	‐	‐	
Ice cream	Citrus fiber: 0%	‐	‐	‐	‐	‐	‐	18,010	82.88	3.55	9.76	Dervisoglu and Yazici, (2006)
	Citrus fiber: 0.4%	‐	‐	‐	‐	‐	‐	18,240	81.73	4.43	12.68	
	Citrus fiber: 0.8%	‐	‐	‐	‐	‐	‐	16,920	81.72	4.15	12.14	
	Citrus fiber: 1.2%	‐	‐	‐	‐	‐	‐	25,780	81.00	4.07	12.23	
Ice cream	Control	704.8 g	‐	‐	‐	‐	‐	42	94.46	−3.05	13.27	Akalın et al., (2018)
	2% apple fiber	620.6 g	‐	‐	‐	‐	‐	133	77.29	4.00	22.04	
	2% orange fiber	1,125.8 g	‐	‐	‐	‐	‐	120	85.40	−1.30	21.62	
	2% oat fiber	621.7 g	‐	‐	‐	‐	‐	43	89.76	−2.60	14.56	
	2% bamboo fiber	727.4 g	‐	‐	‐	‐	‐	71	90.90	−2.55	14.07	
	2% wheat fiber	855.6 g	‐	‐	‐	‐	‐	82	88.76	−2.93	14.16	
Ice cream	Orange fiber: 0%	1,569 g	‐	0.11	‐	208	0.82	‐	76.40	2.29	8.80	Crizel et al., (2014)
	Orange fiber: 1%	2,402 g	‐	0.11	‐	413	0.85	‐	69.18	3.25	13.96	
	Orange fiber: 1.5%	7,294 g	‐	0.10	‐	348	0.87	‐	72.86	3.93	14.08	
Ice cream	Persimmon puree: 0%	16,545 g	‐	‐	‐	−799.0	‐	479.6	70.11	−1.22	4.83	Karaman et al., (2014)
	Persimmon puree: 8%	4,521 g	‐	‐	‐	−422.5	‐	457.0	66.46	1.40	8.09	
	Persimmon puree: 16%	4,751 g	‐	‐	‐	−528.3	‐	278.6	61.38	3.55	12.89	
	Persimmon puree: 24%	5,004 g	‐	‐	‐	−550.8	‐	252.3	55.24	5.07	13.81	
	Persimmon puree: 32%	9,812 g	‐	‐	‐	−595.4	‐	182.0	53.50	5.21	14.70	
	Persimmon puree: 40%	11,372 g	‐	‐	‐	−619.0	‐	141.0	50.56	6.52	17.09	
Ice cream	Quince seed powder: 0%	171 g	‐	‐	‐	‐	‐	266	85.44	−2.38	10.42	Kurt and Atalar, (2018)
	Quince seed powder: 0.25%	178 g	‐	‐	‐	‐	‐	242	82.31	−0.52	9.23	
	Quince seed powder: 0.05%	123 g	‐	‐	‐	‐	‐	262	80.22	0.54	8.80	
	Quince seed powder: 0.75%	99 g	‐	‐	‐	‐	‐	317	78.63	1.22	8.88	
Yogurt	Apple fiber: 0%	‐	‐	‐	‐	‐	‐	‐	97.02	−2.24	12.61	Damian, (2013)
	Apple fiber: 1%	‐	‐	‐	‐	‐	‐	‐	75.76	−0.87	18.34	
Yogurt	Apple pomace: 0%	0.43 *N*	8.18	0.32 N	‐	‐	‐	‐	‐	‐	‐	Wang et al., (2020)
	Apple pomace: 1%	0.47 N	9.15	0.37 N	‐	‐	‐	‐	‐	‐	‐	
	Apple pomace: 2%	0.65 N	12.46	0.52 N	‐	‐	‐	‐	‐	‐	‐	
	Apple pomace: 3%	0.88 N	17.44	0.70 N	‐	‐	‐	‐	‐	‐	‐	
Yogurt	Apple pomace: 0%	1.32 N	30.02	0.80 N	‐	‐	‐	‐	‐	‐	‐	Wang, Kristo, et al., (2019)
	Apple pomace: 0.1%	1.67 N	36.39	1.07 N	‐	‐	‐	‐	‐	‐	‐	
	Apple pomace: 0.5%	2.42 N	39.18	1.36 N	‐	‐	‐	‐	‐	‐	‐	
	Apple pomace: 1%	2.23 N	34.94	1.63 N	‐	‐	‐	‐	‐	‐	‐	
Yogurt	Date fiber: 0%	37.5 g	‐	0.56	‐	−76.1	7.4 mm	‐	95.5	−8.0	9.1	Hashim et al., (2009)
	Date fiber: 1.5%	36.5 g	‐	0.55	‐	−64.0	7.3 mm	‐	84.8	2.7	9.7	
	Date fiber: 3.0%	55.0 g	‐	0.53	‐	−175.7	7.6 mm	‐	80.1	4.1	11.0	
	Date fiber: 4.5%	57.0 g	‐	0.53	‐	−180.4	7.7 mm	‐	75.4	4.9	12.4	
Yogurt	Pumpkin fiber: 0%	‐	‐	‐	‐	‐	‐	‐	95.38	−2.33	11.08	Bakirci et al., (2017)
	Pumpkin fiber: 0.5%	‐	‐	‐	‐	‐	‐	‐	91.00	1.79	20.55	
	Pumpkin fiber: 1%	‐	‐	‐	‐	‐	‐	‐	90.32	2.81	23.25	
	Pumpkin fiber: 1.5%	‐	‐	‐	‐	‐	‐	‐	89.14	3.68	25.30	
Yogurt	Strawberry pulp:0%	‐	‐	‐	‐	‐	‐	‐	82.52	−2.21	‐	Jaster et al., (2018)
	Strawberry pulp:15%	‐	‐	‐	‐	‐	‐	‐	73.40	9.45	‐	
	Strawberry pulp:30%	‐	‐	‐		‐	‐	‐	67.96	15.93	‐	

^a^ L* = lightness; a* = redness (+) and greenness (–); b* = yellowness (+) and blueness (–).

^b^ No data available.

^c^ Newton.

Cape gooseberry is an exotic fruit that attracts great attention because of its nutritional and functional characteristics. It is characterized by plentiful amounts of essential minerals, polyphenols, vitamin C, vitamin A, vitamin B‐complexes, minerals, tocopherol, and carotenoid (Bravo & Osorio, 2016). The effect of cape gooseberry addition at various levels (5%–15%) on the physicochemical and sensory characteristics and mineral content of ice cream was studied (Erkaya et al., 2012). The addition of cape gooseberry in ice cream formulation decreased the fat, protein, pH, and overrun values. At the same time, it improved the total solid, titratable acidity, ash, apparent viscosity values, and first dripping and complete melting times. The sensorial analysis results show that the ice cream contains 15% cape gooseberry had the highest score by the panelists.

## 4 YOGURT

Yogurt is a fermented dairy product obtained by lactic acid fermentation during the action of *Lactobacillus delbrueckii* ssp. *bulgaricus* and *Streptococcus thermophilus*. The resulting lactic acid reacts with milk protein, promoting the characteristic textural and sensorial properties of this product (Serafeimidou et al., 2013). Also, yogurt is rich in protein, fat, calcium, potassium, B vitamins (B_1_, B_2_, B_6_, nicotinic and pantothenic acids) (Hanson & Metzger, 2010; Hashemi Gahruie et al., 2015). Some fruits and vegetables powders have been considered as potential yogurt stabilizing agents due to their desirable functional characteristics, including water binding, gelling, and thickening ability. For example, addition of yam soluble fiber to stirred‐type yogurt increased the apparent viscosity of products and decreased the syneresis of yogurt and produced a suitable mouth feel (Ramirez‐Santiago et al., 2010). Besides, passion fruit by‐products (Espírito‐Santo et al., 2013), apple pomace (Issar et al., 2017; Wang, Kristo, et al., 2019), carrot cell wall particles (McCann et al., 2011), date fiber (Hashim et al., 2009), apple, wheat, bamboo and inulin fibers (Staffolo et al., 2004), carrot juice (Cliff et al., 2013), orange fiber (García‐Pérez et al., 2005), and pineapple peel and pomace powders (Sah et al., 2016) confirmed to improve the structure and decrease the syneresis of yogurts. Sensory quality and acceptability of dairy products fortified with fruits or vegetables were reported in Table 4. The effect of date fiber on the quality properties and consumer acceptance of fortified yogurt was studied by Hashim et al., (2009). The authors reported that yogurt incorporated with up to 3 g of date dietary fiber per 100 g of yogurt had similar sourness, sweetness, firmness, smoothness, and total acceptance ratings as the control yogurt (without added dietary fiber). Still, that sensory ratings and acceptability of yogurt reduced significantly when increasing dietary fiber to 4.5%. In another study, apple pomace extract was used to create a fiber‐enriched acidophilus yogurt, that demonstrated satisfactory sensorial qualities (Issar et al., 2017). The addition of apple pomace as a functional ingredient in yogurt and yogurt drinks was investigated by Wang et al., (2020). Apple pomace added to a diluted yogurt system has the potential to stabilize the acid drink and reduce the sedimentation of protein aggregates. Also, this study demonstrated that apple pomace has potential as a natural stabilizer as well as a dietary fibers source in yogurts and yogurt drinks.

**TABLE 4:** Sensory quality and acceptability^a^ of dairy products fortified with fruits or vegetables

Dairy product	Fruit or vegetable	Appearance	Color	Firmness	Sweetness	Sourness	Flavor	Consistency	Texture	Overall acceptability	References
Cheese	Broccoli: 3%	‐^b^	7.4	‐	‐	‐	6.8	‐	6.33	6.2	Sharma et al., (2011)
	Broccoli: 5%	‐	7.3	‐	‐	‐	7.1	‐	6.6	6.8	
	Broccoli: 10%	‐	7.2	‐	‐	‐	7.2	‐	6.3	6.3	
	Broccoli: 15%	‐	6.7	‐	‐	‐	5.7	‐	5.6	6.0	
	Broccoli: 20%	‐	5.8	‐	‐	‐	5.6	‐	5.7	5.6	
	Broccoli: 30%	‐	4.4	‐	‐	‐	4.8	‐	5.0	3.4	
	Broccoli: 50%	‐	3.1	‐	‐	‐	3.4	‐	4.3	3.2	
Cheese (Queso Blanco)	Tomato extracts: 0%	1.25	‐	‐	‐	1.38	‐	‐	4.25	‐	Jeong et al., (2017)
	Tomato extracts: 0.5%	3.39	‐	‐	‐	2.25	‐	‐	4.39	‐	
	Tomato extracts: 1%	4.54	‐	‐	‐	2.25	‐	‐	4.96	‐	
	Tomato extracts: 1.5%	5.82	‐	‐	‐	2.68	‐	‐	4.96	‐	
	Tomato extracts: 2%	6.11	‐	‐	‐	3.25	‐	‐	5.54	‐	
Ice cream	Citrus fiber: 0%	4.01	‐	‐	‐	‐	7.61	4.00		‐	Dervisoglu and Yazici, (2006)
	Citrus fiber: 0.4%	4.22	‐	‐	‐	‐	8.53	4.37		‐	
	Citrus fiber: 0.8%	4.00	‐	‐	‐	‐	8.13	4.33		‐	
	Citrus fiber: 1.2%	3.63	‐	‐	‐	‐	7.13	3.67		‐	
Ice cream	Orange fiber: 0%	‐	8.12	‐	‐	‐	8.12	‐	8.06	8.10	De Moraes Crizel et al., (2013)
	Orange fiber: 0.74%	‐	7.68	‐	‐	‐	6.96	‐	7.04	7.20	
Ice cream	Orange fiber: 0%	‐	8.0	‐	‐	‐	7.9	‐	7.4	7.9	Crizel et al., (2014)
	Orange fiber: 1%	‐	6.4	‐	‐	‐	6.2	‐	6.3	6.4	
	Orange fiber: 1.5%	‐	6.7	‐	‐	‐	6.5	‐	6.6	6.4	
Ice cream	Quince seed powder: 0%	9.2	‐	‐	‐	‐	7.3	‐	6.7	6.6	Kurt and Atalar, (2018)
	Quince seed powder: 0.25%	8.8	‐	‐	‐	‐	7.3	‐	6.5	6.5	
	Quince seed powder: 0.05%	8.6	‐	‐	‐	‐	7.1	‐	6.9	6.5	
	Quince seed powder: 0.75%	8.4	‐	‐	‐	‐	7.0	‐	8.0	7.3	
Yogurt	Date fiber: 0%	8.3	8.5	7.6	6.9	‐	7.5	‐	‐	7.4	Hashim et al., (2009)
	Date fiber: 1.5%	6.2	6.3	6.7	5.9	‐	6.1	‐	‐	6.8	
	Date fiber: 3.0%	6.4	6.2	6.6	6.2	‐	5.9	‐	‐	6.8	
	Date fiber: 4.5%	6.5	6.2	5.9	5.9	‐	5.1	‐	‐	5.8	
Yogurt	Apple pomace fiber: 0.0%	‐	7.0	‐	‐	‐	6.9	6.7	‐	6.9	Issar et al., (2017)
	Apple pomace fiber: 2.5%	‐	7.2	‐	‐	‐	7.4	7.6	‐	7.3	
	Apple pomace fiber: 5.0%	‐	7.9	‐	‐	‐	8.0	8.4	‐	8.3	
	Apple pomace fiber: 7.5%	‐	3.8	‐	‐		4.3	3.9		3.6	
	Apple pomace fiber: 10.0%	‐	7.0	‐	‐	‐	4.4	3.9	‐	3.8	

^a^ A Nine‐point hedonic scale, 1= dislike extremely, 5= neither like nor dislike and 9= like extremely.

^b^ No data available.

In addition to nutritional value, fiber‐rich fruits and vegetables powder may possess functional properties, including water hydration, fat adsorption, viscosity, and textural properties (Elleuch et al., 2011; Salehi, 2021). The addition of apple fiber in yogurt manufacture reduced the lightness (L*) value and increased a* and b* values (Damian, 2013; Staffolo et al., 2004). Orange fiber (0%, 0.6%, 0.8% and 1% doses and different fiber sizes) obtained from orange juice by‐products was added to yogurt (García‐Pérez et al., 2005). Fiber addition did not cause changes in yogurt acidification and color during the fermentation process, though decreased L* value and increased b* value of the milk. pH decreased and syneresis grew along with cold storage. Yogurt samples with 1% fiber were considerably various from the others along with cold storage, presenting low L* index, high a* and b* index values, and the lowest syneresis. In another study, the utilization of apple pomace fiber (2.5%–10%) in the preparation of fiber‐enriched acidophilus yogurt was investigated by Issar et al., (2017). The apple fiber‐enriched yogurt was found to be the most appropriate concerning the appearance, body/consistency, flavor, and overall acceptability. Thus, apple fiber can be used as a new source of dietary fiber in the food industry. Based on sensory analysis, enriched yogurt containing 5% apple pomace fiber was judged as the best and therefore optimized for the preparation of fiber‐enriched acidophilus yogurt with desirable qualities and sensorial characteristics.

Pumpkin presents a valuable source of some vitamins (A, E, B_1_, B_2_, B_6_, and C), minerals (K, P, Mg, Fe, and Se), β‐carotene, and dietary fibers (Salehi, 2020f; Salehi & Aghajanzadeh, 2020). There are some studies relating the production of yogurt prepared with pumpkin puree or powder, to improve flavor and nutritional value of yogurt (Bakirci et al., 2017; Barakat & Hassan, 2017; El Samh et al., 2013; Jung et al., 2011; Najgebauer‐Lejko et al., 2015; Shin et al., 1993). The effect of pumpkin fiber (0.5%, 1.0%, and 1.5%) on physicochemical, microbiological, rheological behavior and microstructural properties of reduced‐fat yogurt samples (1.55%) was studied by Bakirci et al., (2017). The authors reported that the addition of pumpkin fiber caused a reduction in L* index values (from 94. 9 to 88.7), but caused a raise in a* index (from −2.1 to +4.2) and b* index (from 10.8 to 25.9) values. Also, pumpkin fiber improved the physical quality and contributed textural properties of half‐fat yogurt. The yogurt samples containing 1% pumpkin fiber showed higher storage (G′) (3,687.9 at 21 days) and loss (G″) (543.1 at 21 days) moduli in comparison with other samples. In another study, different vegetable‐fiber enriched yoghurts namely control, pumpkin, carrot, green pea, and zucchini were produced by Yildiz and Ozcan (2019). They reported that vegetable puree supplementation affected the pH, titratable acidity, syneresis, color, texture, and sensorial properties of the yoghurts. Also, firmness, consistency, and viscosity indices were higher in the yoghurt produced with carrot puree, whereas the highest antioxidant capacity was detected in the pumpkin yoghurt, which corresponded to the highest total phenolic, ascorbic acid, and total carotenoid contents.

Carrot is rich in β‐carotene and bears ascorbic acid, thiamine, riboflavin, vitamin B‐complex, tocopherol and it is classified as vitaminized food. It also carries carbohydrates and minerals including calcium, phosphorus, iron, potassium, magnesium, copper, manganese, sulfur, and phenolic compounds (Salehi, 2018; Salehi et al., 2016). Thus, the combination of carrot (juices or by‐products) and yogurt will improve the nutritional and functional food characteristics of the yogurt (Fan & Cliff, 2017; Puvanenthiran et al., 2014). Salwa et al., (2004) have examined the effect of the carrot juice blending ratio on the shelf life and sensorial properties of yogurt and reported that shelf life and panelist acceptance were improved with a 15% carrot juice addition. In another study, Kiros et al., (2016) have studied the effect of carrot juice (0%, 10%, 15%, and 20%) and gelatin stabilizer (0.5%, 0.6%, and 0.7%) on the physicochemical and microbiological characteristics of yogurt. The authors reported that yogurt with suppressed syneresis and improved nutritional and total carotenoids content can be processed from 10%–15% carrot juice and 0.7% stabilizer additions.

Strawberry yogurt is one of the most consumed because of the sensorial characteristics like flavor and color, in addition to the nutritional properties including the presence of bioactive compounds (such as vitamin C, β‐carotene, anthocyanins, and phenolic compounds) (Ariza et al., 2016). The anthocyanin is the main phenolic compounds in strawberry. This compound shows antioxidant and anti‐inflammatory characteristics, also a large variety of chemotherapeutic effects, avoiding the oxidative stress and improving the antioxidant defenses (Aaby et al., 2012; Gasparrini et al., 2017; Jaster et al., 2018). To enhance the functionality and antioxidant capacity of yogurt, some food ingredients such as strawberry were added to the yogurt by some researchers (Estrada et al., 2011; Hanson & Metzger, 2010; Janiaski et al., 2016; Lesme et al., 2020; Lovely & Meullenet, 2009; Oliveira et al., 2015; Silva et al., 2013; Tirloni et al., 2015). Improvement of antioxidant activity and physicochemical characteristics of yogurt supplemented with concentrated strawberry pulp (15% and 30%) was investigated by Jaster et al., (2018). The author reported that the addition of the cryoconcentrated strawberry pulp in the yogurt samples resulted in a product with 3‐fold more anthocyanin content and antioxidant activity. Therefore, the addition of strawberry to the yogurts formulation will improve antioxidant capacity, nutritional and functional food properties of the yogurts.

The intake of dietary fibers and the probiotics exerts a positive impact on the improvement of the intestinal microbiota and is reported to relieve constipation and decrease the incidence of colon cancer (Farnworth, 2008). Furthermore, epidemiological investigations relate the impact of civilization‐induced diseases to insufficient dietary fibers ingestion from fruits and vegetables (Nawirska & Kwaśniewska, 2005). Also, the beneficial effects on probiotics viability exerted by some ingredients such as fruits and vegetables pieces or pulp to dairy products have been examined (Espírito Santo et al., 2010; Kourkoutas et al., 2006; Sendra et al., 2008). Do Espírito Santo et al., (2012) investigated the effect of the supplementation of total dietary fiber from apple, banana, or passion fruit by‐products on the acidity, bacteria counts, and fatty acid profiles in skim milk yogurts co‐fermented by four different probiotics strains. Apple and banana fibers enhanced the probiotic viability during shelf‐life. All the fibers enlarged the short‐chain and polyunsaturated fatty acid contents of yogurts compared to their respective control samples in singular. Also, the effects of fruits and vegetables as a source of dietary fibers on the rheological behavior of yogurt have been examined, and showed stable physicochemical properties of fortified yogurt during storage (Sendra et al., 2010; Staffolo et al., 2004; Tseng & Zhao, 2013). In addition, some studies reported good stability of the bioactive compounds from grape and callus extracts in enriched yogurt (Karaaslan et al., 2011).

## 5 CONCLUSION

Dairy products such as cheeses, ice creams, and yogurts are consumed all over the world and the improvement of these products with vitamins, antioxidants, fibers, and polyphenols may be achieved through the integration of rich sources. The enrichment of dairy products with fruits and vegetables is an effective way to improve the nutritional aspect and to promote functionality by effect on the rheological behavior and physicochemical properties of the final product. In this review, the effects of some fruits and vegetables on the quality of dairy products such as cheeses, ice creams and yogurts have been presented. Fruits and vegetables addition influence the physicochemical and sensory properties of cheeses, ice creams, and yogurts. Some fruits and vegetables fibers proved to be promising food ingredients since they can be used to decrease the fat content and increase bioactive compounds content. Due to the nature of the fruits and vegetables fiber, functionally they hold such properties as increased water binding and holding, thickening, and gelling. Also, fibers from fruits and vegetables improve probiotics viability and increase conjugated fatty acids content in dairy products. Therefore, the fortification of dairy foods with fruits and vegetables could help to provide functional dairy products with high nutritional values and acceptability. Also, fruits and vegetables can be introduced to improve the appearance, color, and attractiveness of fortified cheeses, ice creams, and yogurts for consumers and to increase the sale of these products.

## CONFLICT OF INTEREST

I declare that I do not have any conflict of interest.

## ETHICAL APPROVAL

This study does not involve any human or animal testing.

## INFORMED CONSENT

Written informed consent was obtained from all study participants. The manuscript is not submitted or under consideration in any other journal.

## Data Availability

Data sharing is not applicable to this article as no new data were created or analyzed in this study.
